# AI‐Assisted Decision‐Making for End‐Stage Organ Failure: Opportunities and Ethical Concerns

**DOI:** 10.1111/aor.70027

**Published:** 2025-11-17

**Authors:** John W. Haller, Olga D. Brazhnik, Kathleen N. Fenton

**Affiliations:** ^1^ Advanced Technologies and Surgery Branch, Division of Cardiovascular Sciences National Heart, Lung, and Blood Institute, National Institutes of Health (NIH) Bethesda Maryland USA

**Keywords:** AI, artificial intelligence, decision support, end‐stage organ failure, ethics, organ failure, transplantation

## Abstract

AI holds significant promise for guiding clinical decisions in end‐stage organ failure, where treatment options now include medical management, transplantation, mechanical support devices, and palliative care. This paper discusses current applications of AI in healthcare, emphasizing the complex decision‐making necessary for patients with organ failure. It outlines how AI can support risk stratification, patient selection, and outcome prediction, particularly in transplantation practices that increasingly rely on robust data to inform care pathways. By analyzing large datasets from electronic health records, imaging, and patient‐reported outcomes, AI can help physicians forecast long‐term survival and quality of life, and potentially assist clinicians in modifying treatment strategies before adverse trajectories take hold. There is a need for standardized, high‐quality data, rigorous validation, and transparent algorithms to mitigate biases that could exacerbate disparities in care. Ethical considerations demand attention to equitable access, patient privacy, and the preservation of the human element in patient‐clinician relationships. Patients generally view AI with cautious optimism, recognizing its potential to augment care but also voicing concerns about data protection and the risk of losing compassionate, personal care. Importantly, AI can help with “alignment,” or fitting treatment recommendations to patients' values, and promote sustainable and patient‐centered outcomes. Ultimately, the successful integration of AI into daily practice requires multidisciplinary collaboration among developers, clinicians, ethicists, and regulators. Deep stakeholder engagement, continuous algorithmic refinement, and user‐friendly design are pivotal to ensuring that AI serves as a practical decision‐support tool that complements, rather than replaces, clinical expertise and shared decision‐making.

## Graphical abstract

Artificial intelligence (AI) can help physicians make better decisions for people with serious organ problems by analyzing medical data to predict outcomes. Patients want AI to support, not replace, personal care from doctors. Collaboration among experts ensures AI tools respect patient values and privacy.

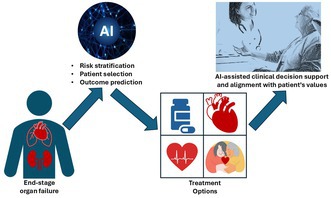

## 1 Introduction

Although end stage organ failure is a common cause of morbidity and death, there is an increasing variety of treatment options [1, 2, 3]. As options become more divergent and more successful, navigating them is becoming ever more complex for patients, families, and health care teams. Patients with heart failure, for example, have traditionally been managed medically, with variable life expectancy and variable quality of life; heart transplantation has been a true game changer for some patients, but its success has been hampered by limited organ availability, and by the almost inevitable onset of infections and rejection that shorten life expectancy. In addition, many patients with organ failure are not good candidates for transplantation (meaning they are unlikely, or are thought to be unlikely, either to ultimately receive an organ, or to do well with it once transplanted). More recently, long term success with mechanical support devices such as left ventricular assist devices (LVADs) has led to their use as destination therapy for patients with heart failure, and total artificial hearts are in clinical trials. Palliative care should also not be overlooked as an option, not only because it may often be best aligned with patients' values, but also because, in some cases, it actually is associated with better life expectancy and quality of life than more “aggressive” management, whether medical or surgical [4, 5].

Each patient with end‐stage organ failure is different. In order to make the best possible decision for an individual patient, the patient, the family, and the entire health care team need to take under consideration all the intricacies of the patient's own medical and social situations, as well as the most updated information on outcomes with each different potential treatment modality, and put this together with the patient's own preferences and values to make an individual and personal recommendation for what pathway would likely yield the outcome that is predicted to be best for that one person. This presents a significant challenge for human decision‐makers, but we hope that with the assistance of Artificial Intelligence (AI) and Large Language Models (LLMs), a giant step could be taken towards it.

## 2 Current Status of AI in Healthcare

Integration of AI into clinical workflow should prioritize responsible AI principles, including transparency, explainability, and explicit human‐in‐the‐loop decision‐making to support, rather than supplant, clinical judgment. AI is transforming healthcare by enhancing patient care, streamlining operations, and driving innovation. Technologies like machine learning, natural language processing, and computer vision are improving diagnostics, treatment planning, and patient outcomes. AI analyzes vast medical data for earlier disease detection, personalized treatments, and predictive analytics. It aids in interpreting medical images with high precision and accelerates drug discovery by identifying drug candidates and optimizing clinical trials. AI‐powered chatbots also enhance patient engagement by providing real‐time health information. However, challenges such as data privacy, the need for quality datasets, and potential biases in AI models must be addressed. AI systems must reflect core bioethical principles [6].

- *Trust* in responsible AI depends on transparency, with clear documentation of algorithmic processes so clinicians and patients understand how predictions are generated.
- *Justice* calls for fair access, bias mitigation, and inclusive design to avoid worsening health disparities.
- *Autonomy* requires that patients and clinicians retain control over decisions, supported by explainable recommendations.
- *Beneficence* compels the use of AI to enhance outcomes and align care with patient goals.

These principles guide ethical development and responsible integration of AI into clinical workflows.

Collaboration between technologists, clinicians, and ethicists is essential to ensure that AI complements healthcare rather than replaces it. As AI adoption accelerates, it is vital to leverage its benefits while maintaining compassion and empathy in medical practice.

## 3 Current Status of AI in Transplant Medicine

AI is increasingly playing a transformative role in transplant medicine by enhancing various aspects of patient care, from diagnosis to postoperative management. AI technologies are improving diagnostics and monitoring, such as using AI‐based electrocardiogram models to detect cardiac allograft rejection, providing a noninvasive screening option [7]. AI helps predict long‐term mortality in kidney transplant recipients using preoperative ECG data [8] and aids in personalizing immunosuppressant dosing in pediatric liver transplants, leading to more precise management [9]. Additionally, AI models improve liver transplantation outcomes by optimizing patient selection [10] and are being integrated into surgical procedures to enhance precision and reduce complications [11]. The use of AI in donor‐recipient matching and patient monitoring facilitates personalized medicine by optimizing drug dosing [9, 12]. AI tools, including chatbots and predictive algorithms, enhance transplant care efficiency through real‐time monitoring and decision support [13, 14]. However, challenges such as data quality, bias, and ethical concerns must be addressed [11, 14]. AI also improves risk stratification and predictive modeling, providing more accurate risk assessments [15, 16] and can revolutionize patient education by offering personalized materials and information, especially in resource‐limited settings [17]. Explainable AI models, such as self‐explaining neural networks, enhance transparency and predictive accuracy for heart transplant survival [18]. Large‐scale algorithms can also predict patient risk [19], supporting Organ Procurement and Transplantation Network's (OPTN) goal of fair, data‐driven, and effective organ distribution.

## 4 What Is Needed for AI to Help Guide Decisions in End‐Stage Organ Failure?

A comprehensive approach will be essential to effectively harness the potential of AI in decision‐making for patients with end‐stage organ failure. The cornerstone of this approach will be the availability of extensive, high‐quality clinical data, encompassing electronic health records, imaging, lab results, demographic information and treatment outcomes [20, 21, 22]. Standardization and interoperability of these data across healthcare systems are crucial for AI algorithms to analyze it effectively, particularly in the context of evaluating treatment options including a variety of medical therapies, devices, transplants and palliative care. Standardization and interoperability of these data across healthcare systems are crucial for AI algorithms to analyze it effectively, particularly in the context of evaluating treatment options including a variety of medical therapies, devices, transplants and palliative care.

AI's predictive analytics could enable the analysis of extensive patient data to predict the progression of end‐stage organ failure and help identify suitable candidates for transplantation, for example. Furthermore, AI could be used to forecast posttransplant outcomes, as well as outcomes of alternative treatment options, aiding clinicians in making informed recommendations about the most appropriate course of action for individual patients, providing personalized treatment recommendations based on individual patient data, taking into account a large number of factors including comorbidities, genetic information, and previous treatment responses. This personalized approach could lead to more effective and tailored treatment plans, ultimately improving patient outcomes. To further enhance decision‐making, AI systems should provide ongoing feedback to update treatment plans based on real‐time patient data and outcomes. Ideally, this would not be at a single point in time but rather could be used to track the patient's progress with a given course of treatment, and suggest a change in plan when warranted, likely long before the patient or clinician would identify the (unfavorable) trajectory.

AI algorithms could help optimize the allocation of donor organs by matching them with suitable recipients based on a multitude of clinical and demographic variables. This would hopefully lead to improved transplant success rates and reduced waitlist times for organ transplants. Still, the development of advanced AI algorithms capable of navigating the complexities of end‐stage organ failure data and providing reliable predictions is a formidable task. Rigorous clinical evaluation and testing in clinical settings are essential to establish the efficacy and safety of these algorithms in guiding treatment decisions, including the assessment of transplant suitability and posttransplant care [20, 21]. AI models must undergo prospective clinical evaluation to ensure their reliability in assisting with treatment decisions. Responsible AI systems should incorporate regular reporting of algorithmic performance and continuous performance monitoring to ensure ongoing accountability and system improvement. Furthermore, continuous monitoring is necessary to evaluate the performance of AI systems and adjust them as medical knowledge and standards of care evolve. Additionally, collaboration with interdisciplinary teams can ensure AI systems remain adaptable and relevant to evolving medical practices and patient needs. Continuous adjustment and updating of AI systems are essential for their evolution within a dynamic “learning health system” [23].

The successful integration of AI into clinical workflow requires seamless alignment with healthcare professionals' practices, particularly in the context of evaluating and managing transplant options. AI algorithms for decision‐making exist on a continuum, ranging from low human involvement to high human involvement. It's important to consider the level of human guidance and assumptions placed on the algorithm [20]. In the context of treatment decisions, AI will not replace clinicians as the decision‐makers but will instead support and enhance the efforts of clinical teams in managing end‐stage organ failure. As more control is ceded to algorithms, it's important to ensure fairness, equitability, and veracity in their decision‐making processes, especially when dealing with treatments for end‐stage organ failure [20, 21]. Responsible AI requires explicit attention to nondiscrimination and prevention of bias throughout model development and deployment to promote equitable care. Adequate training for clinicians on interpreting AI‐generated recommendations while retaining the authority to make final decisions on patient care, including the suitability for and management of organ transplants, will be the imperative.

## 5 Empowering Decisions in End‐Stage Organ Failure

Research suggests that aligning treatment with patients' personal goals and values is crucial for improving uptake and sustainability; it also is an important way to respect the bioethical principle of autonomy, which emphasizes the right of individuals to make informed decisions about their own health care. This involves exploring and acknowledging patients' values, providing access to a variety of care perspectives, and offering values‐aligned health care [24]. The concept of “alignment” has been proposed as a shift from “adherence,” emphasizing the need for patient and clinician to consider various factors that influence treatment implementation [25]. This shift also reinforces ethical imperatives of respecting autonomy and supporting shared decision‐making as a moral good in patient care. Studies have shown that patients often prioritize self‐management and day‐to‐day coping over medical cures; this may clash with healthcare providers' goals [26]. To address this, tools like goal elicitation measures have been developed to support shared decision‐making and align treatment choices with patient values, particularly for surgeries with lasting impacts [27]. These findings underscore the importance of establishing agreed‐upon management strategies that consider both clinical priorities and patient goals. This also supports justice, by ensuring equitable engagement, and beneficence, by focusing on outcomes that matter to patients.

## 6 How Do We Get There From Here?

The first step in developing an AI algorithm to help recommend management of patients with end‐stage organ failure is the collection of high‐quality, comprehensive data. This includes historical outcome data from the different management strategies, clinical outcomes, and, in the case of transplantation, donor and recipient characteristics. Going forward, it could potentially also include genomic, proteomic and metabolomic information as well as data like geospatial coding. The data must be cleaned and preprocessed to ensure accuracy and reliability. Anonymization is crucial to protect patient privacy, especially given the sensitive nature of health data.

Once the data are ready, the next phase involves developing machine learning models. These models need to be capable of predicting outcomes, such as survival rates, graft or device success, and any potential complications, as well as quality of life metrics. Algorithms like logistic regression, random forests, and neural networks could be used, depending on the complexity of the data and the specific outcomes being predicted. The models must be trained on a subset of the data and then tested on another to evaluate their predictive accuracy. When choosing an appropriate machine learning model to train, it is essential to consider interpretability, ensuring that clinicians can understand and trust the model's predictions. Interpretability is particularly important when dealing with complex data and treatment decisions.

Clinical evaluation is essential to ensure the model's reliability and generalizability. This involves rigorous testing using independent datasets and, ideally, prospective validation in clinical settings. The model must demonstrate that it can make accurate predictions across diverse patient populations and treatment scenarios.

For the AI system to be useful, it must be integrated seamlessly into existing clinical workflows. This requires collaboration with healthcare providers to ensure the system supports their decision‐making processes without adding undue complexity. Responsible AI requires explainability, meaning clinicians and patients must be able to interpret and comprehend the rationale behind AI‐generated recommendations. User‐friendly interfaces and clear, interpretable outputs are necessary for clinicians to trust and effectively use the system.

An AI algorithm for clinical decision support must be transparent, with clear explanations for its decisions to ensure trust and accountability. Regulatory approval will also be necessary, requiring adherence to standards set by bodies such as the FDA in the United States. Exactly what type of regulatory approval is appropriate remains an open question, but it certainly involves demonstrating that the AI system is safe, effective, and compliant with patient privacy laws. Public engagement (including experts in medicine and data science) will be needed to determine the best type of oversite for the use of AI in medicine and is likely already overdue.

The field is progressing, but widespread implementation is still a few years away, primarily due to nontechnical challenges. Data availability and quality remain significant barriers, as comprehensive and standardized datasets are essential for training robust AI models. Additionally, ensuring ethical use and securing regulatory approval require careful navigation of complex legal and ethical landscapes. Moreover, the integration of AI into clinical practice necessitates changes in healthcare infrastructure and clinician training, which can be slow processes. Gaining acceptance among healthcare providers and patients is also critical, as they must be confident in the AI system's recommendations. This may ultimately be the most difficult step; as AI systems are further integrated into health care and trust is gradually developed, it will be important to keep in mind the ethical principle of autonomy in relationship to both patients and health care providers, who should (both), to the degree possible, be able to independently decide whether and how to use AI assistance.

In summary, while the technological capability to develop AI algorithms to help manage end‐stage organ failure is advancing rapidly, broader implementation will require overcoming data, ethical, regulatory, and integration challenges. With continued research, collaboration, and investment, AI‐driven decision support systems could become a reality in the next few years, leading to outcomes that are both objectively better and more aligned with patient preferences.

## 7 How Patients Perceive AI in Healthcare and Transplant Medicine

The perception of AI utilization in healthcare varies among patients and is influenced by several factors, including trust, understanding, and perceived benefits and risks. Overall, patients tend to have a cautiously optimistic view of AI in healthcare, seeing its potential benefits but also expressing concerns.

Patients generally display a positive attitude towards AI, particularly in areas where AI can enhance understanding and decision‐making. For example, AI is perceived as useful in interpreting radiology imaging data, preparing for consultations, and confirming doctors' opinions [28]. However, trust is a significant factor influencing acceptance. Patients tend to trust AI more when it is transparent and provides explanations for its predictions [28].

Despite recognizing AI's potential, patients express concerns about losing the human touch in healthcare, data privacy, and the accuracy and empathy of AI systems [28, 29]. These concerns highlight the need for AI systems to be designed with empathy and transparency to foster trust and acceptance.

Patients' perceptions can vary depending on AI's application. For example, in the context of AI voice robots for follow‐ups, patients may be less cooperative if they perceive the interaction as impersonal [30]. Conversely, AI's role in scheduling and administrative tasks is generally well‐received, as it doesn't directly interfere with personal patient‐doctor relationships [29].

Socio‐demographic factors such as age, gender, and education level also influence patient perceptions. For instance, individuals with lower education levels may have more negative perceptions of AI after interacting with AI‐generated content, such as ChatGPT explanations [31].

In conclusion, while patients recognize the potential benefits of AI in enhancing healthcare delivery, significant attention must be paid to addressing their concerns about trust, privacy, and the preservation of the human element in patient care to ensure successful AI integration [28, 29, 32].

As discussed in Section 2, trust in AI depends on transparency, context, and appropriate role assignment between technology and clinicians [33].

## 8 Empowering Patients

AI has the potential to significantly improve clinical decision‐making and to empower patients—especially those living with complex, life‐limiting conditions such as end‐stage organ failure. Patient empowerment involves equipping individuals with the knowledge, tools, and confidence to participate actively in decisions about their care. When properly implemented, AI can help achieve this by translating complex clinical data into insights that are understandable, relevant, and aligned with patient values and preferences.

To be genuinely empowering, AI systems must go beyond risk prediction. They should be capable of synthesizing clinical, psychosocial, and patient‐reported data—including functional status, quality of life metrics, and personal goals—to support shared decision‐making. These systems should facilitate discussions about trade‐offs and treatment options, helping patients and caregivers weigh decisions in the context of what matters most: whether that be longevity, symptom relief, maintaining independence, or engaging in meaningful daily activities.

This approach aligns with core ethical principles such as autonomy, beneficence, and justice. It also reflects the World Health Organization's (WHO) emphasis on health promotion [34] and enables individuals to take greater control over their health. Empowering patients in this way will strengthen evidence‐based medicine—which calls for integrating clinical expertise with the best available evidence and patient values and support public health objectives by fostering informed and engaged health behaviors at a societal level.

Importantly, patient‐centered AI must acknowledge and integrate the broader network of support that shapes care trajectories. Family members, friends, nurses, social workers, and other caregivers—as well as nonhuman agents such as care robots or digital assistants—can offer valuable insights into a patient's needs and preferences. AI systems should be designed to incorporate, or at least prompt the integration of, input from these stakeholders. For instance, AI could analyze communication patterns to flag unmet support needs or encourage timely discussions about advance care planning.

To fulfill their promise ethically and equitably, AI systems must also address barriers related to health literacy, digital literacy, and access. If tools are too complex or fail to accommodate individuals with limited technological experience, they may inadvertently deepen existing health disparities. This underscores the need to bridge the digital divide [35] by ensuring equitable access to technology, designing user‐friendly interfaces, offering culturally responsive education, and supporting ongoing training for both patients and clinicians.

AI systems should be continuously monitored and improved to adapt to new data and evolving healthcare landscapes. Systems must be validated for diverse populations and clinical contexts to remain trustworthy and useful, particularly in high‐stakes areas like transplantation and end‐stage care.

By embracing inclusive design principles and centering the human experience, AI can evolve from a purely analytical instrument to a facilitator of empathy, agency, and human connection. In doing so, it amplifies the patient's voice in decisions that directly affect their life and wellbeing—making healthcare more just, personalized, and compassionate. These efforts echo the ethical priorities of trust, justice, autonomy, and beneficence introduced earlier.

## 9 Ethical Considerations

Although full of promise, the potential use of AI to help guide decision making for patients with end stage organ failure is fraught with ethical issues; it is important to consider and discuss these in parallel with technology development, so that efforts can be made simultaneously to minimize them. These concerns build on the foundational principles outlined earlier, especially justice, autonomy, and trust.

The very fact that AI draws information from existing sources has the potential to introduce bias, and also to magnify any biases or disparities that exist. For example, if a subset of the population does not commonly receive a certain treatment, AI may conclude (incorrectly) that this treatment will not benefit them, creating a positive feedback loop that exacerbates health disparities. Similarly, if some patients are referred (or diagnosed) later and therefore have worse outcomes of any given treatment, the AI may not recommend the treatment based on the demographics. These factors need to be taken into account when data sources are chosen, when algorithms are written, and (perhaps most importantly to clinicians) when recommendations are interpreted.

From a bioethical perspective, this raises concerns related to the principle of justice, as systemic inequities may be reinforced unless explicitly addressed in AI model development and deployment. Another ethical consideration relates to accessibility of AI, should it prove to improve outcomes. In the first place, cost is a consideration: it could be that it will be an expensive system which is only available for patients with significant economic resources. In order to be fair, every effort should be made to develop systems that are inexpensive and broadly available, even in low resource environments domestically and internationally. A related accessibility concern is how hard it is to use the technology: it is important to make sure that it is “user friendly” for both clinicians and the lay public, so that everyone can benefit.

As discussed previously, it may be challenging to fully comprehend the algorithms employed by AI systems in generating their output. The ethical principle of autonomy also reinforces the need for transparency in AI recommendations, so that patients and clinicians can make truly informed decisions. From an ethical standpoint it is important that we have enough information to be sure that they are drawing from good data sources, and that the algorithm is fair to everyone involved. This transparency will allow clinicians, policy makers and the public to have enough confidence to be willing to use these systems. Responsible AI requires robust privacy and confidentiality protections. Ensuring data privacy while allowing AI systems to continually learn from diverse sources will be a significant challenge.

As discussed earlier, trust in AI is multifaceted and depends on clear task allocation, technical transparency, and human oversight. These dimensions must remain central in ethical implementation. Establishing clear roles in human‐AI collaborations will be the key for good use of AI in all aspects of health care, but particularly in cases like end stage organ failure, where decisions are complex. The patient entrusts himself/herself to a physician in a fiduciary relationship, in which the physician is committed to do or recommend what is best for the patient, or to truly care for the patient. This relationship cannot ever be delegated to a computer. AI has a phenomenal potential to analyze huge amounts of information and provide recommendations—but they are just recommendations. AI is a tool that can be immensely helpful for both clinicians and patients, but it needs to be kept “in its lane” as a tool and never become or be considered as a replacement. Words are important: even in the short term we can help remind people of this by the way we phrase or describe things. Pope Francis summoned it up well in a recent address to the G7: “The machine makes a technical choice among several possibilities based either on well‐defined criteria or on statistical inferences. Human beings, however, not only choose, but in their hearts are capable of deciding.” [36].

## 10 Conclusion

Achieving trustworthy AI implementation in end‐stage organ failure care will require consistent alignment with principles from both biomedical ethics and responsible AI frameworks. This includes safeguarding autonomy, beneficence, nonmaleficence, and justice [37], while ensuring transparency, fairness, and accountability in algorithm development and deployment. We reference key responsible AI frameworks, such as the OECD AI Principles [38] and the EU Ethics Guidelines for Trustworthy AI [39], to situate our approach within internationally recognized standards [33].

In conclusion, realizing AI's potential in supporting decisions for end‐stage organ failure treatment, including the evaluation and management of organ transplants, necessitates a concerted effort across data management, technological innovation, ethical practice, regulatory compliance, clinical integration, education, and patient engagement. These elements must harmonize to improve patient outcomes effectively in the context of end‐stage organ failure and transplant care.

## Author Contributions

John W. Haller, Olga D. Brazhnik and Kathleen N. Fenton each contributed to the writing. All authors critically revised the report.

## Disclosure

Disclaimer: The views expressed in this article are those of the author and do not necessarily represent the views of the National Heart, Lung, and Blood Institute, the National Institutes of Health, or the United States Department of Health and Human Services.

## Conflicts of Interest

The authors declare no conflicts of interest.
